# Going Viral: Understanding Medical Misinformation and Older Adults’ Vaccine Hesitancy

**DOI:** 10.1093/geroni/igaa057.1216

**Published:** 2020-12-16

**Authors:** Raza Mirza, James Hull, Siyu Peng, Yalda Mousavi, Muhammad Ibrar Mustafa, Peter Papageorgiou, Eric Rotgaus, Jessica Hsieh

**Affiliations:** 1 National Initiative for the Care of the Elderly, Toronto, Ontario, Canada; 2 National Initiative for the Care of the Elderly, Toronto, Canada; 3 University of Toronto, Toronto, Canada; 4 University of Toronto, Toronto, Ontario, Canada

## Abstract

Influenza persists as a common communicable disease and remains a significant cause of disease burden across the world. Despite preventative therapies, such as influenza vaccination to reduce its spread and transmission, influenza continues to be a source of morbidity and mortality, even in developed countries. For the population over the age of 65, the effects of influenza virus may be more severe when they are compounded by pre-existing conditions and reduced natural immune function. In light of plateauing vaccination rates, a scoping review was conducted to map the literature and determine why seniors aged 65 and above refuse or fail to receive seasonal influenza vaccination. Nine peer-reviewed academic databases covering both social sciences and medical research were searched, along with the grey literature. A total of 6,562 references were identified; after the screening process, 118 references were included in the final review. Thematic analysis focused on the broad areas that positively or negatively influence older adults’ decision-making regarding influenza vaccination, and this resulted in five main themes: (1) barriers to obtaining vaccination; (2) social factors; (3) personal characteristics; (4) individual subjectivity; and (5) direct clinical interventions. This review aims to identify gaps in knowledge and synthesize currently available information to make recommendations for future research, policy development and clinical practice. Increasing the vaccination rate among Canadian older adults will contribute to ongoing efforts to reduce the spread of the influenza virus among the population, reducing influenza-associated hospital admissions and deaths.

